# Sewage and sewage-contaminated environments are the most prominent sources to isolate phages against *Pseudomonas aeruginosa*

**DOI:** 10.1186/s12866-021-02197-z

**Published:** 2021-05-01

**Authors:** Bahareh Lashtoo Aghaee, Mohammadali Khan Mirzaei, Mohammad Yousef Alikhani, Ali Mojtahedi

**Affiliations:** 1 Department of Microbiology, Faculty of Medicine, Hamadan University of Medical Sciences, Hamadan, Iran; 2 Institute of Virology, Helmholtz Center Munich and Technical University of Munich, 85764 Neuherberg, Bavaria Germany; 3 Research Center for Molecular Medicine, Hamadan University of Medical Sciences, Hamadan, Iran; 4 Brucellosis research center, Hamadan University of Medical Sciences, Hamadan, Iran; 5 Department of Microbiology, School of Medicine, Guilan University of Medical Sciences, Rasht, Iran

**Keywords:** *Pseudomonas aeruginosa*, Antibiotic resistance, Phage isolation, Phage therapy, Host-range

## Abstract

**Background:**

*P. aeruginosa* is the primary source of hospital-acquired infections. Unfortunately, antibiotic resistance is growing to precariously high levels, making the infections by this pathogen life-threatening and hard to cure. One possible alternative to antibiotics is to use phages. However, the isolation of phages suitable for phage therapy— be lytic, be efficient, and have a broad host range —against some target bacteria has proven difficult. To identify the best places to look for these phages against *P. aeruginosa* we screened hospital sewages, soils, and rivers in two cities.

**Results:**

We isolated eighteen different phages, determined their host range, infection property, and plaque morphology. We found that the sewage and sewage-contaminated environments are the most reliable sources for the isolation of *Pseudomonas* phages. In addition, phages isolated from hospital sewage showed the highest efficiency in lysing the bacteria used for host range determination. In contrast, phages from the river had larger plaque size and lysed bacteria with higher levels of antibiotic resistance.

**Conclusions:**

Our findings provided additional support for the importance of sewage as the source of phage isolation.

**Supplementary Information:**

The online version contains supplementary material available at 10.1186/s12866-021-02197-z.

## Introduction

*P. aeruginosa* is one of the three bacterial pathogens listed as a critical priority for developing new antibiotics, and a leading cause of nosocomial infection [1, 2]. *P. aeruginosa* causes infections in multiple organs, including skin, respiratory, urinary, and gastrointestinal tracts [3, 4]. Infections by this pathogen are often hard to treat due to the emergence of multidrug resistant strains and the fact that antibiotics are losing their effects [4–6]. Bacterial viruses called phages may provide a solution due to their unique antibacterial characteristics [7–9]. Unlike antibiotics, phages are highly specific, meaning beneficial bacteria stay unharmed during phage treatment [7, 10, 11]. They are also ubiquitous- they outnumber their bacterial host in most ecosystems. The abundance of virus-like particles (VLPs)— which also include phages —in the environment can range from high: ~ 10^9^ g^− 1^ in wetlands to low: ~ 10^3^ g^− 1^ in hot deserts in addition to the ~ 10^9^ m^− 2^ VLPs, which are deposited from the atmosphere every day [12–14]. Thus, it should be relatively easy and cheap to isolate phages from the environment against multidrug resistant bacteria compared to developing new antibiotics [9, 15]. However, not all phages are good for therapeutic applications [7, 11]. One limitation of using phages as antibacterial agents is their narrow host range— many phages can only infect a few bacterial strains. Therefore, to fight against the high diversity of bacteria that can cause infection, we would need large numbers of distinct phages [7, 9, 15].

Under existing regulation, all phages must undergo an extensive clinical trial before being applied as antibacterial agents [7, 16, 17]. Thus, using phages with a broader host range can limit the number of phages required to treat infections by different bacterial pathogens, which reduces the cost of clinical trials [15–17]. Using phages with a broad host range could also lead to fewer treatment failures due to an unsuitable phage-host matching when a ready-to-use approach is followed [9, 17, 18]. For phage therapy, most phages are isolated from different environments including, sewage, freshwater, soil, and more— normally where the bacterial hosts exist [9, 15]. These sources contain the high number of VLPs [13, 14] with the highest reported for sewage ~ 10^10^ ml^− 1^ [19], suggesting them as prominent sources for phages isolation. Yet, they differ markedly with respect to physical conditions like temperature, and nutrient availability, which can affect phages-bacteria interactions, and result in selection of phages with specific infection properties [20–24]. Recent metagenomic studies indicated that broad host range phages, infecting several species to multiple phyla, are widely distributed in natural environments [20, 25–27]. Nonetheless, these findings are mainly based on in silico predictions and need further experimental validations. There is also some evidence supporting the existence of broad host range phages— can infect many strains of one species or lyse multiple bacterial species [7, 15, 28, 29]. Taken together, these show the possibility of isolating broad host range phages against different target bacteria from the environment. It will be, therefore, of great interest to identify environmental sources with higher chances to isolated broad host range phages against bacteria with high priority to developing new antibiotics against like *P. aeruginosa*.

To this end, we screened three environmental sources where *P. aeruginosa* is commonly present: sewage, soil, and river [30, 31]. We isolated 18 Pseudomonas phages from these environments in different cities, Rasht and Hamadan, in Iran. We next studied their host range using the efficiency of plating (EOP) test and determined the infection properties, latency period, and burst size of three phages. We then analyzed the correlation between the source of isolation and multiple phage characteristics (host range, EOP values, plaque size, plaque morphology, latency period, and burst size) and bacterial features (antibiotic resistance and genetic variation). We demonstrated that phages isolated from different sources tended to have distinct characteristics and infected bacteria with different features.

## Materials and methods

### *P. aeruginosa* strains

Twelve MDR strains with different genomic features, and resistant profiles were selected from a *P. aeruginosa* collection previously isolated from burn wound patients and used in the study for phage isolation [32]. This study was conducted following the Institutional Review Board-approved studies: IR.UMSHA.REC.1396.923, Hamadan University of Medical Sciences.

### Isolation of phages

We sampled three distinct environmental sources, including hospital sewage, river, and soil from two different cities: Rasht (37° 16′ 33″ N, 49° 35′ 19″ E) and Hamadan (34° 47′ 57″ N, 48° 30′ 52″ E). These cities are 360,6 km away and have a different climate. Hamdan is a mountain city with a height of 1800 m above sea level. In contrast, Rasht is located close to the Caspian Sea, and it is 8 m above sea level. From these two cities, we collected a similar number of samples per city/source, a total of eighteen, from two hospitals, three rivers, and soil from multiple landscapes. The environmental samples were isolated from both contaminated and uncontaminated sites. The collected samples screened for phages using *P. aeruginosa* collection, previously isolated and characterized from burn patients [32]. Specifically, 50 mL of the water collected from rivers and sewages were centrifuged at 4000×g for 30 min and sterile filtered to remove biological matter. Soils were mixed with phosphate-buffered saline (PBS) at a 1:10 ratio, weight to volume (w/v), and thoroughly vortexed, centrifuged, and sterile-filtered to separate phages from the organic matter [1, 7, 33]. The filtrates were mixed with equal amounts of double-strength LB and 10 mL of an overnight culture of the target bacteria and incubated for 18 h at 37 °C. Next, 10 ml of the incubated mix was centrifuged at 4000×g for 15 min and sterile-filtered through a 0.45-μm membrane filter. The filtered cultures were tested for phages using a standard plaque assay. Isolated phages were re-isolated by plaque purification from the LB agar plates when multiple phages on the same plate were suspected [1, 7].

### Host range determination

We used Efficiency of Plating (EOP) to determine the host range of the isolated phages [7]. Twelve bacterial strains to be tested were grown at 37 °C until they reached OD: 0.08 at 625-nm, equivalent to 1.5 × 10^8^ CFU/ml. We used 200 μl of each culture together with 100 μl of diluted phage lysate, 10^6^–10^9^ times dilutions from the phage stock, in double-layer plaque assays. All three replicates for each phage were done in parallel on the bacterial strains tested in this study. The plates were incubated for 18 h at 37 °C, and the number of plaque-forming units (PFU) was counted for each combination. The EOP was calculated (average PFU on target bacteria / average PFU on host bacteria) and presented together with the standard deviation [7] for the three measurements (Table S1 as supplementary file).

### One-step growth experiment

The host bacteria were grown to the exponential phase and infected by phages at the multiplicity of infection of 0.1 and incubated with shaking at 37 °C. Next, 500 μL of samples were collected every 10 min, centrifuged, filter-sterilized through a 0.45-μm membrane filter, and kept on ice until titration by plaque assay [1, 34]. The latency periods were calculated as the difference between the time of phage inoculation and the time of the release of phage progenies [34]. Burst sizes were calculated by dividing the average phage titers of the time points after the burst from the initial average of infecting phage titers [1, 34]. The infection property of three phages, vB_PaeM_GUMS6, vB_PaeM_GUMS32, and vB_PaeM_GUMS45, were taken from our earlier study [1].

### Statistical analysis

The statistical analyses were conducted in the R open-source software [35]. The *stats* package was used to run all ANOVAs and t-tests. *ggplot2* package was used to create the map [36]. *FactoMineR* package [37] was used to run the multiple factor analysis (MFA). *psych* package [38] was used to test the degree of correlation between the quantitative variables using Pearson’s correlation coefficient.

## Results and discussion

### The lowest number of phages against *P. aeruginosa* were isolated from soil

We isolated eighteen phages from eighteen samples collected from three different environmental sources- eight samples from rivers, six from sewages, and four from soil. Fifty percent of phages were isolated from the river samples- more than two phages per sample on average. In contrast, soil contained lowest number of phages against *P. aeruginosa*- less than one phage per sample on average. Similarly, previous studies on soil isolated ~ 100 phages against *Streptomyces avermitilis*, a soil bacterium, from 700 samples that were screened [39]. Soil represents one of the most diverse ecosystems on earth, with an interacting community of bacteria, archaea, viruses, fungi, and protozoa [40]. In addition, thousands of bacterial species are living on soil, including many human pathogens, suggesting soil as a viable source for phage isolation [41]. Yet, soil contains a lower number of VLPs ∼10^8^ g-1 compared to fresh water and sewage, which might partially explain our results.

### Sewage contaminated sites harbored more phages compared to uncontaminated sources

In addition to uncontaminated environmental sources, we screened a river and a farm contaminated with human wastes to see if the chance of isolating phages against *P. aeruginosa* is higher in those. The large majority of phages isolated from environments were from the contaminated sites. Sewage is suggested as a reliable source for isolating phages against aerobic pathogens due to the high abundance of bacteria associated with humans in them [9, 42]. Sewage collects material from a large human population, which significantly expands the diversity of microbes living in these systems, which explains the higher number of phages found in environmental sites contaminated with the human waste [43]. However, the number of phages isolated from the sewage-contaminated river was higher than hospital sewages. This came as a surprise as we were expecting more phages to be isolated from hospital sewages than a local river. One possible explanation could be the difference in type of waste going into these sources. The polluted river collects sewage from different origins, including industry, hospitals, farms, and residential areas, while hospital sewages only receive waste from hospitals. Thus, the contaminated river may host a more diverse *P. aeruginosa* community and its phages, as this bacterium can also be found in other sources [44]. Similarly, isolation of phages against multiple human pathogens from Ganges, one of the most polluted rivers in the world, have been previously reported [45, 46]. In addition, we failed to isolate *Pseudomonas* phages from the uncontaminated river. The reason might be the lower abundance of bacteria from class *Gammaproteobacteria*, that includes *Pseudomonadaceae* family, compared to other taxa that are more abundant like members of phylum *Actinobacteria* [47–49].

**Fig. 1:**
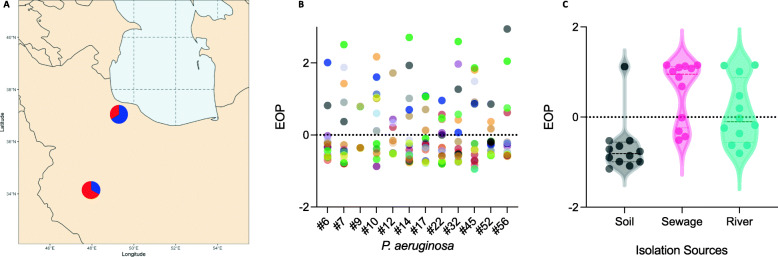
**a** Map showing the fraction of phages isolated at different sites. The ratio of phages isolated on each site is in blue. Pie diagrams at 37° 16′ 33″ N, 49° 35′ 19″ E shows Rasht and 34° 47′ 57″ N, 48° 30′ 52″ E indicates Hamadan. **b** *Z* scores of EOP values of different phages on *P. aeruginosa* isolates- different colors represent different phages. **c** Z scores of phages’ EOP values— grouped based on their isolation source. Sewage phages showed the highest EOP values against bacteria used for host range determination

We also isolated different numbers of phages from the two cities in the study. Specifically, two-third of the phages were isolated from Rasht in North of Iran (Fig. 1a). There could be multiple reasons for this, but the higher incidence of *P. aeruginosa* infection cannot be one of them as the limited studies explored this suggested otherwise [50, 51]. The higher number of phages isolated from Rasht can be partially explained by the higher population density of this city, 180/km^2^ vs. 137/km^2^, which can result in more microbial pollution [52]. The differences between these cities’ climates may also play a role as higher microbial diversity is predicted for humid subtropical climates compared to the semi-arid climates [53, 54].

### Most isolated phages make the non-halo clear plaques with different sizes

From eighteen isolated phages, only three are halo, and four are forming turbid plaques. Plaque sizes range from 1 mm to 4.5 mm, with phages isolated from river tend to make larger plaques on average. Plaque morphology is often used for selecting phages for further characterizations. They can provide information on the phages’ replication cycle, the family they might belong to, and if they encode specific enzymatic activities. Specifically, temperate that can choose between lytic and lysogenic cycle typically produces turbid plaques, in contrast to virulent phages form clear plaques [9, 34]. The family of phages can also be roughly predicted from their plaque morphology. For example, it has been suggested that phages with larger heads like those from the *Myoviridae* family tend to make smaller plaques [9, 55]. Moreover, the semi-transparent zones surrounding phage plaques called halo suggest that the phage might encode extracellular polymeric substances (EPS) depolymerase with potential activity against biofilms [9, 55].

### Phages isolated from sewage had higher EOP values

Next, we aimed to determine the host range of the isolated phages using the EOP method. The phages’ host range were varied between 10 to 12, with 7 out of 18 phages lysed all 12 bacteria used for host range determination (Fig. 1b and c). Isolated phages showed distinct EOP values on different *P. aeruginosa* strains (Fig. 1b and c). The values were also significantly varied from phage to phage, with sewage phages showing the highest efficiency of plating (two-way analysis of variance [ANOVA], Tukey’s post hoc test, *p* < 0.05). For phage therapy, it is highly desirable to use phages that show high virulence on a large number of bacterial strains— an EOP value ≥1 on a target bacterium suggest an efficiency higher or equal to the original host of isolation [7].

### Multiple factor analysis of the interactions among phages infection properties, host characteristics, and the source of phage isolation

**Fig. 2:**
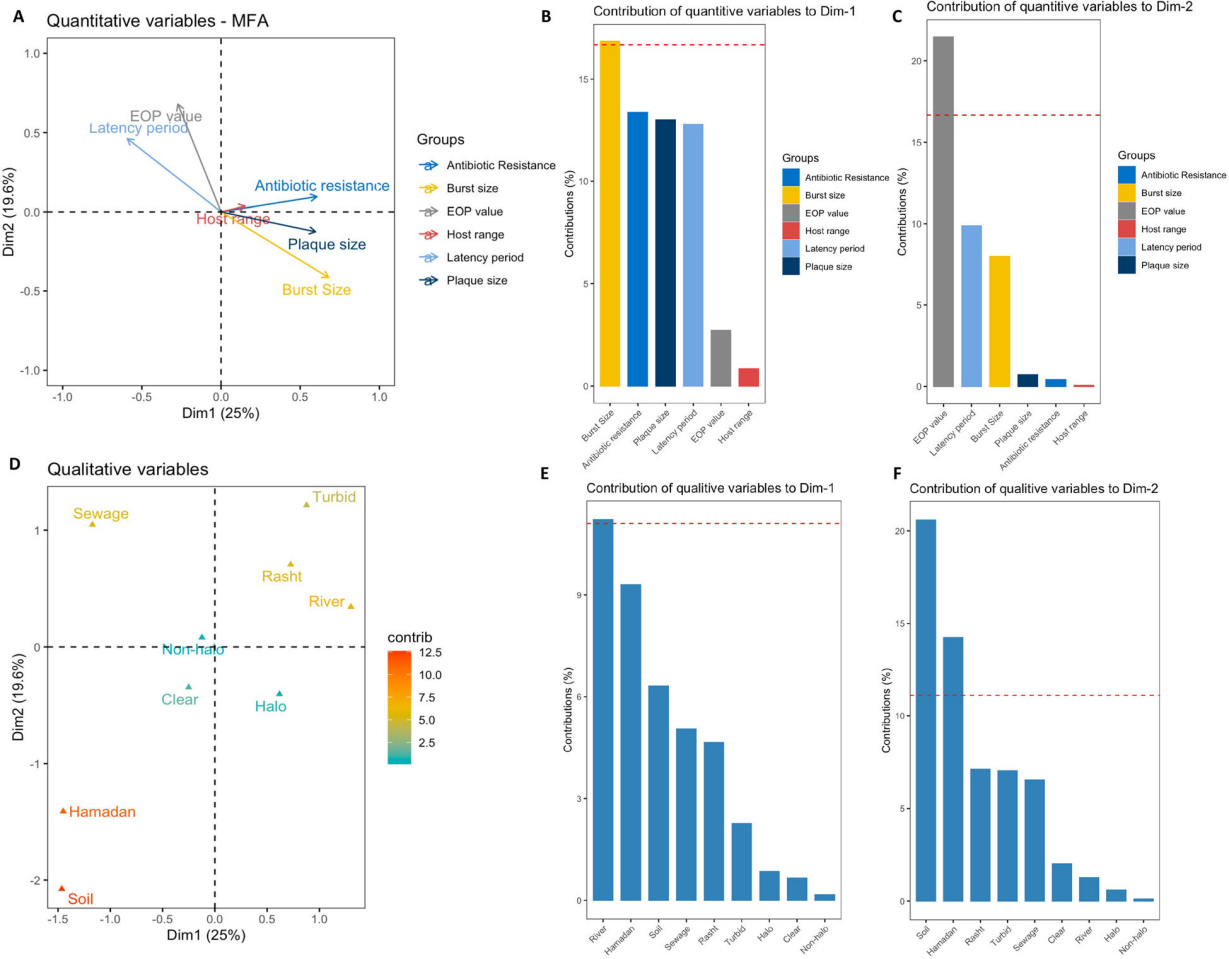
Multiple Factor Analysis of the phages host range, infection property, source of isolation, and bacterial host characteristics. **a** MFA ordination of quantitative variables. **b** and **c** The contribution of each quantitative variable to dimensions 1 and 2 of the MFA. **c** MFA ordination of qualitative variables. **e** and **f** The contribution of each qualitative variable to dimensions 1 and 2 of the MFA. Quantitative variables include antibiotic resistance, plaque size, host range, EOP value, phages latency period, and burst size- quantitative data were scaled. Qualitative variables include plaque morphology, source and location of isolation, and Pulsed-field Gel Electrophoresis (PFGE) type of the host

We also examined the correlations among multiple phage features (host range, infection properties and plaque morphology), host characteristics (antibiotic resistance pattern, and genetic variation), and sources and geographical region where phages were isolated from using a multiple factor analysis (MFA) framework [33, 56]. This allowed assessing the contribution of sources and geographical regions of isolation to different phage and host features. The first dimension of the MFA explained 25% of the total variation in the burst size, antibiotic resistance, and plaque size (Fig. 2a, b, and c), and separated variables according to their correlation with phage plaque morphology, and source of isolation (Fig. 2d, e, and f). The second dimension of the MFA explained an additional 19.6% of the total variability in the EOP value, and latency period (Fig. 2a, b, and c), and largely separated variables correlated to the source of isolation (Fig. 2d, e, and f). In light of these two axes, we found that phages isolated from sewage tended to have higher EOP values and longer latency period, whereas phages isolated from river infected bacteria that were resistant to more antibiotics; they also had larger plaques. In addition, isolated phages could be clustered based on the geographical region, plaque clarity (clear vs. turbid), plaque type (halo vs. non halo), and sources of isolation (Soil, Sewage, and River). The phages isolated from river were more associated with Rasht, whereas phages isolated from soil were linked to Hamadan.

**Fig. 3:**
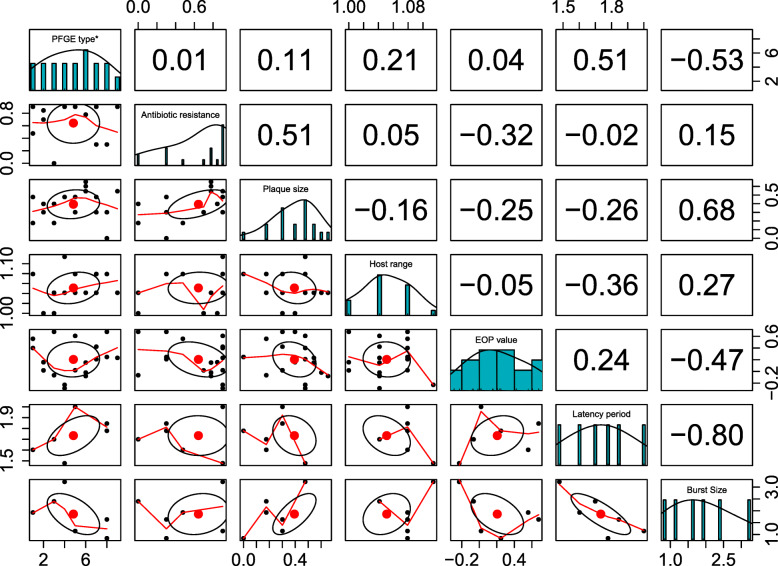
The correlation matrix of quantitative variables. Pearson’s correlation coefficient was used to measure the correlation between different quantitative variables (PFGE type, antibiotic resistance pattern, plaque size, host range, EOP value, latency period, and burst size). The diagonal shows distribution of each variable: on the top, the values of the correlations are displayed, while on the bottom, the bivariate scatters plots with a fitted line are shown

We also ran Pearson’s correlation coefficient analysis of the quantitative variables. The EOP values were negatively correlated to host range, plaque size, antibiotic resistant pattern, yet positively correlated to latency period and PFGE type (Fig. 3). As previously suggested, there is a negative correlation among phages’ infection property- latency period and burst size- and the high EOP values, implying a trade-off between phages’ replication rate and host range [7, 24]. However, our data for the latency period and the burst size is limited and only include six phages, which makes it difficult to precisely evaluate the trad-off, this has been observed in earlier studies. For example, when phages T1, T4, and ϕX174, were tested against an *E. coli* collection, ϕX174 showed the fastest replication rate but narrowest host range among phages tested [24].

## Conclusions

*P. aeruginosa* is unquestionably one of the most successful pathogens, causing severe nosocomial infections with a high risk of morbidity and mortality in patients [2, 6, 57]. Infections by this pathogen are becoming harder to treat as conventional antibiotics are less effective [58]. Phages may provide a solution- they are host specific, ubiquities, and are constantly developing different strategies to attack bacteria [18, 59]. Yet, for a phage to be suitable for therapeutic applications, it should be lytic, have a broad host range, and be highly efficient against target bacteria [7, 15, 34]. Phages are typically found in their natural habitats where their hosts are [9, 60]. However, these habitats may vary in their physical and chemical features, affecting phages-bacteria interactions [54] and selecting for phages with different infection properties. Thereby, it would be helpful to identify environments with a higher probability to contain desirable phages for phage therapy to facilitate the isolation process.

We screened multiple environmental sources in two different cities for isolating phages against *P. aeruginosa*. The samples used in the study were collected from both contaminated and uncontaminated sites. The vast majority of phages were isolated from sewage or sewage-contaminated sources, suggesting human waste as a prominent source for phages isolation against *P. aeruginosa*. These phages also showed the highest EOP values, further emphasizing the importance of sewage as a source for phage isolation. However, we didn’t observe a significant difference in the host range of phages isolated from different environment. In addition, we found that phages isolated from the polluted river had larger plaque size and infected bacteria that were resistant to more antibiotics. Yet, our sample size was limited, and we only screened three different sources. To further evaluate the validity of our findings, future studies by including samples with higher heterogeneity (e.g., samples from human, animal, and marine systems) are warranted. With the growing acceptance of phage therapy as an alternative to antibiotic treatment and the increasing demands for phages against different target bacteria, identifying best places to look for specific phages will facilitates phage isolation against emerging pathogenic bacteria.

Going forward, we are planning to determine the kinetics of resistance development in target bacteria against isolated phages. This will help to identify phages with less probability to grow resistant against using OmniLog [9, 61]. This is especially important as, like antibiotics, bacteria can grow resistant to phages. Thus, selecting phages with less likelihood of developing resistance against for phage therapy, will improve the treatment outcomes by preventing the resistant mutants from developing.

## Supplementary Information

**Additional file 1 Table S1.** The efficiency of plating (EOP), for the eighteen phages on different *P. aeruginosa* isolates. EOP values are presented together with the standard deviation for the three measurements. Data of phages’ plaque morphology, infection property, source of isolation, and hosts’ antibiotics resistance, and genetic variation are included. The antibiotic resistance section shows the number of distinct antibiotics that the different *P. aeruginosa* strains are resistant to. The average EOP values were used for statistical analysis. vB: viruses of bacteria; Pa: *P. aeruginosa*; NA: not applicable; 0: No plaque was seen; 1: Both host and target bacteria are the same; PFGE: Pulsed-field Gel Electrophoresis.

## Data Availability

The datasets used and/or analyzed during the current study available from the corresponding author on reasonable request.

## Abbreviations

*P. aeruginosa*: *Pseudomonas aeruginosa*
VLPs: Virus-like particles
EOP: Efficiency of plating
MDR: Multidrug-resistant
PBS: Phosphate-buffered saline
CFU: Colony-forming units
PFU: Plaque-forming units
EPS: Extracellular polymeric substances
MFA: Multiple factor analysis
